# Assessing Textbook Outcome After Implementation of Transversus Abdominis Release in a Regional Hospital

**DOI:** 10.3389/jaws.2022.10517

**Published:** 2022-07-06

**Authors:** Johannes A. Wegdam, Dite L. C. de Jong, Tammo S. de Vries Reilingh, Ellis E. Schipper, Nicole D. Bouvy, Simon W. Nienhuijs

**Affiliations:** ^1^ Elkerliek Ziekenhuis, Helmond, Netherlands; ^2^ Maastricht University Medical Centre, Maastricht, Netherlands; ^3^ Catharina Hospital, Eindhoven, Netherlands

**Keywords:** complications, transversus abdominis release, complex ventral hernia repair, learning curve, textbook outcome

## Abstract

**Background:** The posterior component separation technique with transversus abdominis release (TAR) was introduced in 2012 as an alternative to the classic anterior component separation technique (Ramirez). This study describes outcome and learning curve of TAR, five years after implementation of this new technique in a regional hospital in the Netherlands.

**Methods:** A standardized work up protocol, based on the Plan-Do-Check-Act cycle, was used to implement the TAR. The TAR technique as described by Novitsky was performed. After each 20 procedures, outcome parameters were evaluated and new quality measurements implemented. Primary outcome measure was Textbook Outcome, the rate of patients with an uneventful clinical postoperative course after TAR. Textbook Outcome is defined by a maximum of 7 days hospitalization without any complication (wound or systemic), reoperation or readmittance, within the first 90 postoperative days, and without a recurrence during follow up. The number of patients with a Textbook Outcome compared to the total number of consecutively performed TARs is depicted as the institutional learning curve. Secondary outcome measures were the details and incidences of the surgical site and systemic complications within 90 days, as well as long-term recurrences.

**Results:** From 2016, sixty-nine consecutive patients underwent a TAR. Textbook Outcome was 35% and the institutional learning curve did not flatten after 69 procedures. Systemic complications occurred in 48%, wound complications in 41%, and recurrences in 4%. Separate analyses of three successive cohorts of each 20 TARs demonstrated that both Textbook Outcome (10%, 30% and 55%, respectively) and the rate of surgical site events (45%, 15%, and 10%) significantly (*p* < 0.05) improved with more experience.

**Conclusion:** Implementation of the open transversus abdominis release demonstrated that outcome was positively correlated to an increasing number of TARs performed. TAR has a long learning curve, only partially determined by the technical aspects of the operation. Implementation of the TAR requires a solid plan. Building, and maintaining, an adequate setting for patients with complex ventral hernias is the real challenge and driving force to improve outcome.

## Introduction

Major surgeries in an aging population maintain the surgical epidemic of incisional hernias (1, 2). Repairing these hernias remains fraught with complications, especially if a patient need a component separation technique (CST) for primary fascial closure (3–6). Such complex abdominal wall repair procedures can be challenging, require well-organized peri-operative multidisciplinary guidance and a team which should be able to adopt new techniques (7).

Latest alteration in component separation techniques is the transversus abdominis release (TAR) (8–15). This posterior CST was introduced in 2012 as an alternative to the classic anterior component separation technique (Ramirez) (8, 16). The TAR is also a myofascial release intended to decrease midline tension, but has an improved overlap of large defects and hernias near bony structures. The safe plane in which the mesh is positioned and lack of extended subcutaneous dissection are also assets (17, 18). Because TAR seemed to have less surgical site occurrences and recurrences than Ramirez, TAR became popular in many hernia centers over the world (10, 11, 19, 20).

The operation itself is described as technically difficult, requiring an intimate understanding of pertinent anatomy to avoid TAR pitfalls (12, 14, 21) (Supplementary Table S1). Division of incorrect layers lead to neurovascular lesions, semilunar hernias, interparietal herniation, and recurrences. Multiple authors mentioned a learning curve of the TAR and advised implementation only after adequate training and proctoring of the first 5–15 cases, depending on the experience in open Rives-Stoppa repair (9–15).

In 2016, a team of surgeons from a regional hospital in the Netherlands commenced with the TAR after attending a TAR workshop with hands-on cadaveric dissections. This study aims to describe the outcome and learning curve of TAR, after implementation of this new technique in a dedicated hernia center.

## Material and Methods

### Setting

The Elkerliek Hospital in Helmond, the Netherlands, is a non-teaching regional hospital with three experienced hernia surgeons performing 75 complex ventral hernia repairs per year. Before the TAR was implemented, endoscopic anterior CST and open Ramirez were standard techniques for complex hernia patients.

### Study Design

The Plan-Do-Check-Act (PDCA) cycle, or *Demming* cycle, was used to implement TAR and repeatedly evaluate outcome (22). PDCA is a four-step problem-solving process involving *plan* (establishing the processes needed to deliver results according to the desired outcome), *do* (implement the new process on a small scale), *check* (measure the new process and observe any differences between that and the desired outcome), and *act* (analyze the difference between observed and expected to determine the cause). The iterative nature of repeated PDCA cycles is critical prerequisite of value-based healthcare (23, 24). In this study, *plan* compromised a standardized work up protocol for each complex hernia patient and continuous registration of at least 200 characteristics per patient in a database. *Do* was implementation of the TAR. Outcome was *checked* after each episode of 20 procedures. Specific measurements to improve outcome were defined and subsequently implemented (*act*). The effect of these measurements was checked again after the next 20 procedures, new measurements were developed and the cycle repeated itself (Supplementary Table S2). All patients consented with the TAR and postoperative data analysis. The Institutional Review Board approved this review.

### Standardized Work-Up Protocol

All eligible patients were informed, both orally and digitally by the patient journey app. After consent, each patient with a symptomatic complex ventral hernia was presented at a monthly multidisciplinary team (MDT) meeting, involving experienced hernia surgeons, anesthetist, ICU physician, pulmonologist, physical therapist and case manager. Patients were discussed according a four-step protocol: (I) hernia was graded according the EHS and the Hernia Patient Wound staging system (25–27). According the Dutch guideline for incisional hernias, a complex ventral hernia is any hernia HPW stage II-IV (28, 29). Hernias <10 cm width were also included if a primary fascial closure could not be achieved without an additional component separation technique, like hernias located against a bony structure or hernias with a significant loss of domain (LOD) > 20% (25, 26, 28, 30). LOD was assessed by the Sabbagh method (30, 31). Parastomal hernias were classified by the EHS parastomal hernia classification (32); (II) surgical options were discussed. Patients with a lateral hernia or midline hernia that passed the semilunar line were initially selected; (III) potential modifiable factors for prehabilitation were identified and feasible goals that had to be achieved for the patient were assessed. Active counseling was provided to ensure a BMI <30 kg/m^2^, smoking cessation more than 4 weeks prior to surgery, glycemic control for diabetics and an optimal mental, physical, cardiopulmonary and nutritional status (5, 33). Pre-operative Botulinum was not applied (34).; (IV) the *decision* was made to plan an operation, postpone surgery until the prehabilitation goals were met or waive any operation.

### Standardized TAR Technique

**FIGURE 1:**
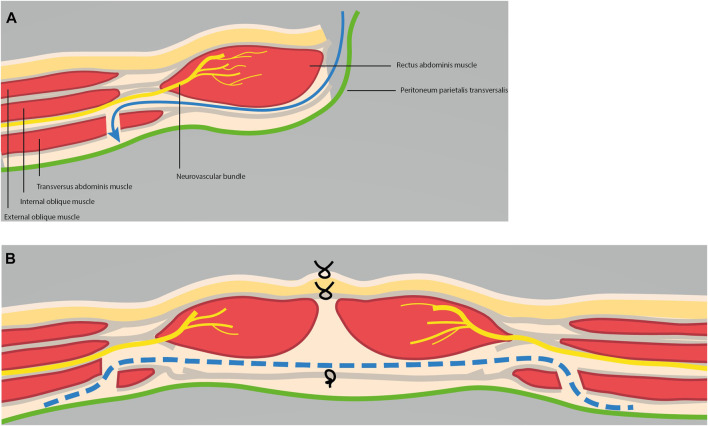
**(A)** Schematic overview of posterior component separation technique with transversus abdominis release. **(B)** Final situation after TAR with retromuscular, preperitoneal mesh in place.

Each patient was operated by two surgeons. Prophylactic antibiotics were administered. Midline laparotomy with excision of the scar was followed by resection of the hernia sac and complete reduction of bioburden, including formerly implanted meshes. A complete enterolysis between bowels and parietal peritoneum was performed. The rectus sheath was then incised approximately 0.5–1 cm from its medial border exposing the rectus muscle and posterior rectus sheet. This retromuscular plane was extended to the retroxyphoidal space superior and the space of Retzius inferior. Laterally, the plane was extended to the linea semilunaris until the neurovascular bundles were visualized medially. To preserve these perforators, 0.5–1 cm medial from the neurovascular bundles, the posterior lamel of the musculus obliquus internus (MOI) was incised exposing the transverse muscle (TM) in the upper abdomen and the inserting fascia of the TM in the lower abdomen. The transversus abdominis fascia and muscle were then subsequently transected exposing the underlying peritoneum/transversalis fascia (PTF). The next step was dissecting the TM from the PTF by sharp and blunt dissection, creating a large plane bordered by the lateral edges of the psoas muscle, retroxyphoidal space and Retzius’ space (Figure 1A). Defects in the PTF that could not be closed, were managed with omentum or an inlay dual layer mesh (Ventralite ST™, BD). After complete posterior CST, the medialized posterior rectus sheaths were then re-approximated with a running slowly resorbable 2/0 monofilament (small bites and steps). A large mesh was placed in retromuscular position between the fasciae and selectively secured anteriorly with two slowly absorbing 2/0 monofilament stitches. The preferred mesh was a permanent large pore monofilament polypropylene mesh (30 × 30 cm Soft Mesh™, BD) in CDC wound class 1-2 or a long-term bioresorbable monofilament Poly-4-hydroxybutyrate mesh (40 × 20 cm or 30 × 25 cm Phasix™, BD) in case of contaminated surgical fields (CDC wound class 3-4), at the surgeons discretion (35). Closed-suction drains were placed laterally on the mesh (8, 21, 36). The anterior rectus sheaths were reapproximated with a running slowly resorbable 2/0 monofilament. Subcutaneous tissue was closed with an absorbable polyfilament running suture (Figure 1B). A subcutaneous drain was placed at the surgeons discretion. Skin was closed intracutaneously with rapid absorbable monofilament and a sterile adhesive plaster and abdominal binder were applied. After 6 weeks of wearing a binder day and night in combination with reduced activities, a protocolized rehabilitation program under guidance of a physical therapist was commenced.

### Outcome Measures

Primary outcome measure is Textbook Outcome (TO): the rate of patients with an uneventful clinical postoperative course after TAR. Textbook Outcome is defined in this study by a maximum of 7 days hospitalization without any complication (wound or systemic), reoperation or readmittance, within the first 90 postoperative days, and without a recurrence during follow up. While comparison of complication rates between hernia studies is biased by registration and interpretation issues, Textbook Outcome enables a comprehensive summary of simple and unambiguous clinical care parameters. Textbook Outcome is used in other surgical specialties for both internal quality improvement and comparison with other studies (37, 38). The number of patients with a Textbook Outcome compared to the total number of consecutively performed TARs is depicted as the institutional learning curve. The institutional learning curve of applying TAR for complex abdominal wall hernias is not equivalent to the surgical TAR learning curve, defined by a minimum number of operations needed for a surgeon to master TAR.

Secondary outcome measures were the details and incidences of the surgical site and systemic complications within 90 days, as well as long-term recurrence and bulging rates. Wound complications were grouped under surgical site occurrences (SSO) and surgical site events (SSE) (39). SSE are all SSIs and clinically relevant SSO. SSOPI are SSO requiring a Procedural Intervention, like percutaneous drainage, wound opening, debridement, negative pressure wound therapy (NPWT) or mesh removal. Seromas and hematomas were subcategorized according the Morales-Conde classification into incidental seromas/hematomas (present max 6 months) or complicated seromas/hematomas (>6 months with discomfort or complications that needed intervention) (40). Complications were graded by the Clavien-Dindo classification system (I-V): severe complications are type IIIb, IVa, IVb and V(41). A recurrence was defined as any new protrusion of the contents of the abdominal cavity or preperitoneal fat through a defect in the abdominal wall at the site of a previous repair of an abdominal wall hernia (42). Postoperative bulging is a bulge in the area of previously repaired hernia. In case of a suspected recurrence, clinical evaluation and CT were always performed.

### Statistics

Continuous variables are presented as mean (SD) and categorical variables by number (proportion). To evaluate the differences between the two independent groups, for continuous variables the Mann-Whitney U Test was used and for categorical variables the Fisher’s exact test. A *p* < 0.05 (two-tailed) was considered statistically significant. Statistical analysis was performed using Microsoft Excel and IBM SPSS Statistics 27.

## Results

During five and a half years (1 January 2016 to 1 July 2021), 491 consecutive complex hernia patients were discussed at the MDT meetings, of whom 289 patients (59%) were ultimately operated. A total of 132 (46%) patients underwent an elective CST: 69 TAR, 57 endoscopic anterior CST (ECST) and 6 Ramirez. Over the years, the rate of anterior CST decreased gradually to zero and TARs increased to 100%.

The baseline characteristics and HPW stages of the 69 TAR patients were distributed per group of 20 consecutive patients and demonstrated no relevant differences between these groups (Table 1). Comorbidity according the HPW classification (P1) was present in 29% of all patients. Pre-operative contamination of the surgical field (W1) was present in 33% of the patients, due to a stoma (*n* = 21), an ulcerated skin (*n* = 1) or open mesh (*n* = 1). Two-third (61%) of the patients were first referred for prehabilitation. Ten (14%) patients had stage I (HPW “non-complex,” <10 cm) hernias, but still needed TAR because of location against the xiphoid or iliac crest, and/or a LOD>20%. Over the years, patients tended to be older (*p* = 0.07), but had less diabetes (*p* = 0.06) and less stoma-related procedures (*p* = 0.14).

**TABLE 1:** Demographics of patients that underwent a TAR.

Episode	I	II	III	IV	Total	*p*-value
N	20	20	20	9	69	
Hernia factors						
Previous incisional hernia repair, n (%)	5 (25)	6 (30)	7 (35)	2 (22)	20 (29)	0.788
Previous wound infection, n (%)	13 (65)	6 (30)	10 (50)	3 (33)	32 (46)	0.932
Hernia location						
Midline (EHS M1-4, L0), n (%)	10 (50)	13 (65)	15 (75)	5 (56)	43 (62)	0.823
Lateral (EHS M0, L1-4)	1 (5)	2 (10)	0 (0)	3 (33)	6 (9)	
Mixed (EHS M1-4, L1-4), n (%)	9 (45)	5 (25)	5 (25)	1 (11)	20 (29)	
Stoma present (including Bricker), n (%)	7 (35)	5 (25)	6 (30)	3 (33)	21 (30)	0.490
Presence of a concomitant parastomal hernia	4 (20)	2 (10)	5 (25)	3 (33)	14 (20)	0.391
Parastomal hernia with concomittant midline hernia (EHS type III/IV)	4 (20)	1 (5)	4 (20)	1 (11)	10 (14)	0.472
Planned concurrent abd. procedure	7 (35)	4 (20)	2 (10)	3 (33)	16 (23)	0.245
Hernia width on CT (cm), mean (SD)	12.3 (4.9)	12.7 (5.6)	13.3 (4.2)	11.0 (4.2)	12.5 (4.8)	0.698
H1: 0-9.9 cm, n (%)	5 (25)	6 (30)	2 (10)	3 (33)	16 (23)	0.238
H2: 10-19.9 cm, n (%)	13 (65)	11 (55)	17 (85)	6(67)	47 (68)	
H3: >20.0 cm, n (%)	2 (10)	3 (15)	1 (5)	0 (0)	6 (9)	
Area of herniaa (cm^2^), mean (SD)	153.0 (112.0)	140.3 (120.8)	157 (105.0)	98.0 (85.8)	143.3 (109.0)	0.267
Loss of domain >20%, n (%)	7 (35)	3 (15)	4 (20)	2 (22)	16 (23)	0.503
Loss of substance, n (%)	9 (45)	7 (35)	8 (40)	3 (33)	27 (39)	0.122
Patient factors						
Age (years), mean (SD)	62.6 (11.6)	58.5 (8.5)	62.5 (11.0)	69.6 (6.3)	62.3 (10.5)	0.073
Males, n (%)	11 (55)	11 (55)	11 (55)	4 (44)	37 (54)	0.819
Oncological history, n (%)	5 (25)	5 (25)	12 (60)	3 (33)	25 (36)	0.090
ASA class III, n (%)	6 (30)	4 (20)	1 (5)	2 (22)	13 (19)	0.364
COPD GOLD I-IV, n (%)	4 (20)	4 (20)	7 (35)	1 (11)	16 (23)	0.851
Cardiovascular disease	7 (35)	5 (25)	7 (35)	2 (22)	21 (30)	0.811
Use of oral anticoagulants, n (%)	9 (45)	7 (35)	6 (30)	4 (44)	26 (38)	0.921
BMI (kg/m2), median (SD)	29.5 (3.2)	27.3 (3.1)	27.6 (3.8)	28.9 (4.9)	28.2 (3.6)	0.200
Obesity (BMI>30 kg/m^2^), n (%)	9 (45)	3 (15)	4 (20)	2 (22)	18 (26)	0.198
P1: Morbid obesity (BMI>35 kg/m^2^)	1 (5)	0 (0)	0 (0)	2 (22)	3 (4)	
P1: Current smoker past 4 weeks, n (%)	0 (0)	0 (0)	4 (20)	1 (11)	5 (7)	
Former smoker	15 (75)	13 (65)	14 (70)	4 (44)	46 (67)	0.432
P1: Diabetes, n (%)	7 (35)	2 (10)	0 (0)	4 (44)	13 (19)	0.060
P1: Use of Immunosuppression, n (%)	1 (0)	0 (0)	3 (15)	0 (0)	4 (6)	
At least one P1 factor present, n (%)	8 (40)	2 (10)	5 (25)	5 (56)	20 (29)	0.189
Wound factors						
W1: CDC wound class 2–4, n (%)	7 (35)	5 (25)	7 (35)	4 (44)	23 (33)	0.367
Pre-operative HPW stage				(0)		
I H1P0W0	3 (15)	5 (25)	2 (10)	0 (0)	10 (14)	0.415^a^
II H1P1W0; H2P0-1W0	10 (50)	9 (45)	11 (55)	4 (44)	34 (49)	
III H1-2P0-1W1; H3P0W0	5 (25)	4 (20)	6 (30)	5 (56)	20 (29)	
IV H3P1W0; H3P0-1W1	2 (10)	2 (10)	0 (0)	1 (11)	5 (7)	
Patients referred for prehabilitation	11 (55)	9 (45)	15 (75)	7 (78)	42 (61)	0.154

TAR (Posterior component separation technique with), Transversus Abdominis Release; EHS, European hernia society; ASA, American society of anesthesiologists; COPD, chronic obstructive pulmonary disease; BMI, body mass index; CDC, center of disease control; HPW, hernia patient wound classification (H1, H2 or H3; P0 or P1; W0 or W1).

a Stage I and II, versus stage III and IV.

Table 2 demonstrates the monthly caseload, partially influenced by the Covid pandemic in latter episodes. The rate of contaminated surgical fields increased during surgery from 33% to overall 42% of the patients, due to 6 (W0) patients that had unintended bowel lesions (4) or an unexpected infected mesh that was explanted (2). Other intra-operative characteristics demonstrated no significant differences, except for the application of topical microporous polysaccharide hemospheres (MPH) (Arista™, Absorbable Surgical Hemostat, BD) to prevent hematomas and seromas, which commenced after the 31st patient. Mean operation time reduced after 60 TARs by half an hour.

**TABLE 2:** Intra-operative characteristics of patients that underwent a TAR.

Episode	I	II	III	IV	Total	*p*-value
N	20	20	20	9	69	
Time span (months)	31	11	13	11	66	
Caseload per month	0.6	1.8	1.5	0.8	1.0	
Contaminated surgical field, n (%)	8 (40)	7 (35)	10 (50)	4 (44)	29 (42)	0.805
Planned concurrent abdominal procedure (stoma reversal or replacement), n (%)	7 (88)	4 (57)	2 (20)	3 (75)	16 (55)	
Unintended contamination of the surgical field (complete bowel lesions), n (%)	3 (15)	3 (43)	1 (10)	1 (25)	8 (28)	0.728
Extirpation of an infected mesh, n (%)		1 (14)	3 (30)	1 (25)	5 (17)	
Blood loss (ml), mean (SD)	103 (151)	240 (483)	184 (425)	184 (335)	176 (372)	0.718
Bilateral TAR performed, n (%)	17 (85)	15 (75)	14 (70)	5 (56)	51 (74)	0.389
Synthetic mesh, n (%)	16 (80)	17 (85)	18 (90)	8 (89)	59 (86)	0.825
Complete anterior fascial closure, n (%)	19 (95)	19 (95)	19 (95)	9 (100)	66 (96)	0.911
Use of topical MPH (powder)	0 (0)	10 (50)	16 (80)	6 (67)	32 (46)	**0.005** ^**a**^
Drain placement, n (%)	10 (50)	8 (40)	4 (20)	2 (22)	24 (35)	0.184
Operation time (min), mean (SD)	186 (84)	174 (56)	180 (42)	160 (52)	178 (61)	0.751

TAR (Posterior component separation technique with), transversus abdominis release; MPH, microporous polysaccharide hemospheres, ^a^ Period I and II versus period III and IV.

bold + p-value <0.05.

The rate of patients with one, or more, systemic complication (48%) was higher than patients with any wound complication (41%). Pneumonia (28%), ileus (14%) and anemia (14%) were most frequent (Table 3). No mortality was noted. SSO and SSI demonstrated a tendency to decrease (respectively, *p* = 0.07 and 0.08) and SSE significantly decreased in the different episodes (*p* < 0.05). Two thirds of all seromas and half of all hematomas were complicated. Eight patients developed a SSOPI (12%) of whom four patients (7%) were reoperated. During the first episode, two patients needed wound debridement (one reoperation, one outpatient), one patient underwent a mesh explant (reoperation) and one patient local excision of exposed synthetic mesh (outpatient, after 82 days). During the second episode one patient needed wound debridement (outpatient) secondary to an unnoticed bowel injury (that spontaneously healed) and one patient underwent mesh explant (reoperation) secondary to an abdominal compartment syndrome. In this patient the posterior fascia could be closed again and a biosynthetic mesh placed on top. The anterior rectus fascia was left open and negative pressure wound therapy was applied. In both the third, and in the fourth episode, one patient each needed wound debridement (one reoperation, one outpatient). Application of MPH did not reduce the rate of seromas (*p* = 0.53) or hematomas (*p* = 0.14) significantly. In none of the patients, intraparietal herniations or semilunar hernias were noted. Length of hospital stay decreased from twelve to 7 days (*p* = 0.16). Readmissions occurred due to wound problems in three patients or constipated stomas in two. Recurrence rate was 4%: all three cases were related to contaminated surgical fields and use of biosynthetic meshes. No iatrogenic semilunar hernias or intraparietal herniations were encountered. Bulging occurred in five patients (7%) and all were laterally located. Three of the six lateral hernias (one with a Bricker) bulged, one mixed hernia bulged laterally after a previous ipsilateral Ramirez and in one patient mixed hernia bulged due to a pre-existent absent unilateral rectus muscle. One year mortality rate was 1% (cerebrovascular attack 11 months after TAR) and two-year mortality rate 4% (another 2 patients died after 19 and 22 months due to oncological causes).

**TABLE 3:** Short (90-day) and long term complications of patients that underwent a TAR.

Episode	I	II	III	IV	Total	*p*-value
N	20	20	20	9	69	
Wound morbidity						
Patients with any SSO, n (%)	12 (60)	9 (45)	4 (20)	3 (33)	28 (41)	0.072
Seroma type I-IV, n (%)	5 (25)	4 (20)	2 (10)	2 (22)	13 (19)	0.659
Surgical Site Infection (SSI), n (%)	7 (35)	3 (15)	1 (5)	1 (11)	12 (17)	0.079
Hematoma type I-IV, n (%)	5 (25)	2 (10)	2 (10)	1 (11)	10 (14)	0.494
Wound dehiscence, n (%)	6 (30)	1 (5)		1 (11)	8 (12)	
Enterocutaneous fistula, n (%)	1 (5)				1 (1)	
Patients with SSE, n (%)	9 (45)	3 (15)	2 (0)	2 (22)	16 (23)	**0.045**
Patients with SSOPI, n (%)	4 (20)	2 (10)	1 (5)	1 (11)	8 (12)	0.517
Systemic complications, n (%)	13 (65)	10 (50)	6 (30)	4 (44)	33 (48)	0.173
Pneumonia, n (%)	6 (30)	5 (25)	6 (30)	2 (22)	19 (28)	0.957
Paralytic ileus, n (%)	4 (20)	2 (10)	3 (15)	1 (11)	10 (14)	0.825
Anemia requiring blood transfusion, n (%)	5 (25)	3 (15)		2 (22)	10 (14)	0.127
Decompensatio cordis, n (%)	2 (10)	1 (5)			3 (4)	
Abdominal compartment syndrome, n (%)		1 (5)			1 (1)	
Maximal Clavin-Dindo classification						
IIIb	2 (10)	1 (5)			3 (4)	
Iva	2 (10)				2 (3)	
Ivb		1 (5)			1 (1)	
Reoperation <90 days, n (%)	2 (10)	2 (10)	1 (5)		5 (7)	
Length of hospital stay (days), mean (SD)	11.2 (9.3)	8.5 (6.2)	7.5 (3.5)	7.2 (3.8)	8.9 (6.6)	0.164
Readmission, n (%)	1 (5)		1 (5)	2 (22)	4 (6)	0.169
Follow up (months), median (SD)	37.0 (12.0)	28.2 (4.8)	23.3 (3.5)	12.3 (3.7)	27.2 (10.7)	
Recurrence, n (%)	2 (10)	1 (5)			3 (4)	0.548
Bulging, n (%)	1 (5)	1 (5)		3 (33)	5 (7)	0.398

SSO, surgical site occurrence; SSE, surgical site event, SSOPI SSO, requiring Procedural Intervention.

bold + p-value <0.05.

**FIGURE 2:**
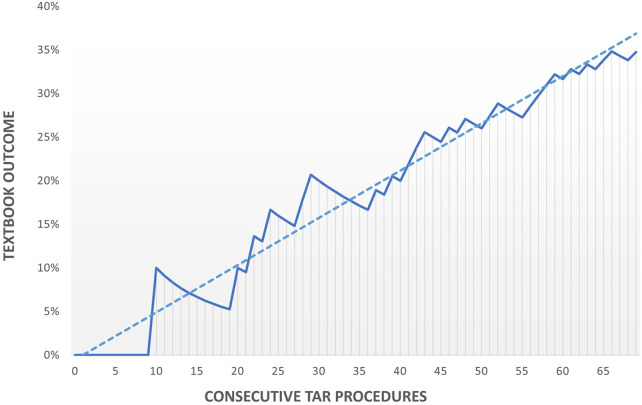
Institutional learning curve of applying TAR for complex abdominal wall hernias.

Contamination of the surgical field was positively correlated to the development of SSOPI (*p* = 0.01). Pre-operative HPW stage was not significantly correlated with any of the outcome parameters. A significant (*p* = 0.01) increase in patients with a Textbook Outcome was found over time (Table 4). After the second episode (40 TARs), Textbook Outcome increased to 55%. The institutional learning curve of TAR demonstrated a gradient of 0.5 and was still rising after 69 procedures (Figure 2).

**TABLE 4:** Textbook Outcome of patients that underwent a TAR.

Episode		I	II	III	IV	Total	*p*-value
n		20	20	20	9	69	
1	Hospital stay ≤1 week, n (%)	7 (35)	11 (55)	13 (65)	6 (67)	37 (54)	
2	No Surgical Site Occurrences <90 days, n (%)	8 (40)	11 (55)	16 (80)	6 (67)	41 (59)	
3	No systemic complications <90 days, n (%)	7 (35)	10 (50)	14 (70)	5 (56)	36 (52)	
4	No reoperations <90 days, n (%)	18 (90)	18 (90)	19 (95)	9 (100)	64 (93)	
5	No readmission <90 days, n (%)	19 (95)	20 (100)	19 (95)	7 (78)	65 (94)	
6	No recurrence during follow up, n (%)	18 (90)	19 (95)	20 (100)	9 (100)	66 (96)	
Textbook outcome (all 6 items present), n (%)		2 (10)	6 (30)	11 (55)	5 (56)	24 (35)	**0.012**

bold + p-value <0.05.

### Check and Act: Evaluation of Outcome (TO, SSO, SSE, SSOPI) and Quality Measurements Implemented

#### Outcome Evaluation After the First Episode of 20 Patients

Textbook Outcome was 10% and the rate of wound complications twice compared to other TAR studies. TAR implementation developed slowly and rate of contaminated fields was high (40%).

Measurements: 1) decreasing contaminated surgical fields by expanding the indication for TAR to include more midline hernias and hernias near bony structures, 2) decreasing SSO by improving prehabilitation (sticking more tight to the predetermined goals, in particular the requirement to have a (BMI <30), 3) increasing the number of monthly complex hernia repair slots and, 4) decreasing hematomas and seromas by increased attention for meticulous dissection in combination with the application of MPH in flanks, on the mesh and subcutaneously.

#### Outcome Evaluation After the Second Episode of 20 Patients

Textbook Outcome increased to 30%, SSE rate decreased from 45% to 15% (*p* = 0.04), SSO and SSOPI rates also decreased (n.s.). The rate of systemic complications (50%), especially pneumonias, remained high. Although more midline hernias were included, contaminated surgical fields did not decrease (35%). Median BMI decreased, monthly case load tripled, and MPH was applied. ACS developed in one patient. Measurements: 1) Reevaluation of the operative protocol: consultation with anesthesiologists led to measuring of the pulmonary plateau pressures under deep neuromuscular block (confirmed by post-tetanic-count stimulation), just before and immediately after midline closure. An arbitrary increasement of ≥6 mm Hg may increase the risk of postoperative pulmonary failure or ACS and could alter the operative strategy from midline closure with an augmenting mesh to a bridging procedure. 2) Reduce drain placement while using MPH application.

#### Outcome Evaluation After the Third Episode of 20 Patients

Textbook Outcome increased to 55% and rate and severity of both wound and systemic and complications was further reduced: SSO to 20% (*p* = 0.03), SSE 10% (*p* = 0.02), SSOPI 5% (*p* = 0.07), despite contaminated fields in 50%. Referrals for prehabilitation increased to 75%. Lack of ICU-capacity due the Covid pandemic severely decreased caseload. Drain placement minimized from 50% to 20%. Rate of systemic complications decreased from 65% to 50%–30% in the third cohort (*p* = 0.08). Severe complications (Dindo > IIIa) did not occur. Measuring the pulmonary plateau pressures did not alter any operative strategy, nor did it reduce the rate of pulmonary infections (30%). Measurement: 1) decrease the rate of patients that need a postoperative ICU bed. The respiratory risk score, developed by Fischer, predicts the risk of postoperative respiratory failure after CAWR (43). This score was implemented to enhance the MDT decision for the need of ICU beds after CAWR. 2) Expand the indication for TAR to giant isolated flank hernias.

## Discussion

Five years after implementing the TAR in our hospital, Textbook Outcome occurred in 35% of 69 consecutive TAR patients. More patients (47%) developed systemic complications, than wound complications (41%). Separate analyses of three comparable cohorts of each 20 consecutive TAR patients demonstrated that both Textbook Outcome (10-30-55%) and clinical relevant wound complications (45-15-10%) significantly improved over time. After 69 TARs, the institutional learning curve of performing TARs for complex abdominal wall hernias still did not flatten.

### Strength and Limitations

The strength of this conclusion is based on the “real world” design of this study: all consecutive TAR patients were included and peri-operative characteristics and complications were recorded meticulously. A strict protocol to select and prehabilitate complex hernia patients was used, a dedicated multidisciplinary team was present and the hospital was equipped with three experienced hernia surgeons. Repeated evaluations (PDCA cycle) generated a deeper insight in the dynamics of different outcome parameters during the course of this study, which helped in defining and implementing new quality measurements.

This study is limited by its retrospective nature. Also, quality of life, perhaps the most important outcome parameter in complex hernia surgery, was yet not evaluated in our patients (15). Pre-operative Botulinum, which might have increased the rate of primary fascial closures or reduced the overall rate of CST, was still not standardized within our protocol (34, 44). This study was not powered to demonstrate that HPW stratification would lead to significant differences in outcome per stage, or to detect variables (like MPH) that may improve outcome independently.

### Evaluation of Outcomes

Our results were compared to similar publications from single institutions, that also reported on their initial TARs (maximum 100 patients) (8, 11, 13, 15, 20, 45–49). Three studies described the rate of patients without any *wound* complications during hospitalization, which was 61–76% (45–47). After the initial 40 TARs in this study, Textbook Outcome increased to 55% in the next 20 patients. This approaches these rates, although those studies did not take a 90-day inclusion period, systemic complications, re-admissions or recurrences into account. Wound complications reported in comparable studies (SSO 3-39%, SSE 3-14%, SSOPI 3-12%) resemble the results reported here, except for SSE (23%), which was high in the first episode (8, 11, 13, 15, 18, 20, 45–49). While SSE is underreported in most studies, and no specific cause can be designated, this may be related to the TAR learning curve (8, 13, 15, 48, 49). The rate of patients without any systemic complication cannot be deduced in any other study, nor can it be adequately compared with our results. Reported rates of recurrences (0–6%) parallel our results (4%).

Larger cohort studies or data from national registries, varying from 184 to 3109 TAR cases, demonstrated slightly better outcomes than the smaller series: SSO 18–31%, SSE 19%, SSOPI 5-9% and recurrence 3-4% (6, 10, 18, 19, 50, 51), which may be due to some learning curve effect.

The finding that more systemic, than wound, complications were noted in our series is interesting, especially in the light of 42% contaminated surgical fields. Increased attention for prehabilitation may have positively affected the SSO rate. The high rate of former smokers (67%) and COPD (23%), a higher rate of forced primary midline closure after TAR that leads to intraabdominal hypertension, a low threshold to register complications, or the fact that underreporting of systemic complications is common in TAR publications, may also have played a role in this high rate of systemic complications (11, 20, 45).

### Learning Curve

The previously reported learning curve to master TAR (around ten) correlates with our SSE rate being the highest in our first episode, more specifically, in the first ten patients (9, 12). However, TAR-specific complications, like damage to the perforating neurovascular bundles, non-closable peritoneal defects, extreme lateral (paracolic) enterolysis leading to unintended bowel injuries with fecal spillage, or primary closure under too much tension leading to an abdominal compartment syndrome, occurred mainly in our first 40 patients. In our experience, mastering the TAR technique may indeed require 5–10 procedures, but understanding for whom the TAR is the best solution, requires more than 10 TARs, and an extensive experience in mastering other component separation techniques as well.

Several authors have emphasized that the real challenge in complex hernia surgery is adequate patient selection (10, 12, 52, 53). Maloney demonstrated in 775 CST patients, that 168 ‘ideal’ patients (BMI <35, not diabetic, no history of smoking, synthetic mesh used, complete fascia closure and a noncontaminated field) had a SSO rate of 21%, compared to 39% in 607 ‘non-ideal’ patients (*p* < 0.05) (10). This not only demonstrates that CST has a high SSO rate and that the institutional learning curve will never be 100%, but also that outcome may be improved by converting “non-ideal” patients into “ideal” patients, possibly by effective prehabilitation (54) Centralization of hernia surgery (52, 53, 55), prehabilitation of modifiable factors like BMI, smoking behavior or physical condition (54, 56–58), building multidisciplinary teams (7), assessing the quality of life by analyzing short- and long term patient-reported outcomes (13, 15, 49), are all quality measurements that improve patient selection and outcome. Thus, the continuous inclination of our straight-lined institutional learning curve, even after 69 TARs, does not only reflect our technical development, but also the improved capabilities in patient preparation and selection.

### Future

There seems a commendable trend in hernia literature to present peri-operative data more precisely (11, 13, 15). Still, interpreting outcome between hernia studies remains comparing “apples to oranges” (42, 59). This can be improved by the unambiguous variable “Textbook Outcome.” Textbook Outcome is easy to understand for patient and health care workers and proved to be a valuable and simple tool to monitor the learning curve. To the best of our knowledge, this study is the first in hernia literature using Textbook Outcome, a simple, powerful and positive parameter. Therefore, future studies describing (new) operative techniques might consider using Textbook Outcome as a function of the learning curve, to put a technique in a broader perspective and make results more comparable. ‘Significantly improved quality of life’ should also become an important element in a new definition of Textbook Outcome.

## Conclusion

The five-year results after implementing the open transversus abdominis release in a regional hospital are presented. Outcome was positively correlated to an increasing number of TARs performed. TAR demonstrated to have a long learning curve, only partially determined by the technical aspects of the operation. Implementation of the TAR in a regional hospital is feasible, but requires a solid plan. Building, and maintaining, the adequate setting for patients with these complex ventral hernias is the real challenge and driving force to improve outcome.

## Data Availability

The raw data supporting the conclusion of this article will be made available by the authors, without undue reservation.
